# Bacteriocin-Based Synergetic Consortia: a Promising Strategy to Enhance Antimicrobial Activity and Broaden the Spectrum of Inhibition

**DOI:** 10.1128/spectrum.00406-21

**Published:** 2022-02-16

**Authors:** Samira Soltani, Eric Biron, Laila Ben Said, Muriel Subirade, Ismail Fliss

**Affiliations:** a Food Science Department, Food and Agriculture Faculty, Laval University, Quebec, Quebec, Canada; b Faculty of Pharmacy, Laval University and Laboratory of Medicinal Chemistry, CHU de Québec Research Center, Quebec, Quebec, Canada; c Institute of Nutrition and Functional Foods, Laval University, Quebec, Quebec, Canada; Pennsylvania State University

**Keywords:** bacteriocins, reuterin, synergy, antimicrobials, combinations, antibiotic resistance, food preservatives

## Abstract

Bacteria-derived natural antimicrobial compounds such as bacteriocins, reruterin, and organic acids have recently received substantial attention as food preservatives or therapeutic alternatives in human or animal sectors. This study aimed to evaluate the antimicrobial activity of different bacteria-derived antimicrobials, alone or in combination, against a large panel of Gram-negative and Gram-positive bacteria. Bacteriocins, including microcin J25, pediocin PA-1, nisin Z, and reuterin, were investigated alone or in combination with lactic acid and citric acid, using a checkerboard assay. Concentrations were selected based on predetermined MICs against Salmonella enterica subsp. *enterica* serovar Newport ATCC 6962 and Listeria ivanovii HPB28 as Gram-negative and Gram-positive indicator strains, respectively. The results demonstrated that the combination of microcin J25 + citric acid + lactic acid; microcin J25 + reuterin + citric acid; and microcin J25 + reuterin + lactic acid tested against *S.* Newport ATCC 6962 showed synergistic effects (FIC index = 0.5). Moreover, a combination of pediocin PA-1 + citric acid + lactic acid; and reuterin + citric acid + lactic acid against *L. ivanovii* HPB28 showed a partially synergistic interactions (FIC index = 0.75). Nisin Z exerted a partially synergistic effect in combination with acids (FIC index = 0.625 -0.75), whereas when it was combined with reuterin or pediocin PA-1, it showed additive effects (FIC index = 1) against *L. ivanovii* HPB28. The inhibitory activity of synergetic consortia were tested against a large panel of Gram-positive and Gram-negative bacteria. According to our results, combining different antimicrobials with different mechanisms of action led to higher potency and a broad spectrum of inhibition, including multidrug-resistance pathogens.

**IMPORTANCE** Reuterin and bacteriocins, including microcin J25, pediocin PA-1, nisin were produced and purified with >90% purity. Using the broth-based checkerboard assay the interaction between these compounds (synergetic, additive, or antagonistic) was assessed. By combining different natural antimicrobials with different modes of action and structure (reuteirn, microcin J25, pediocin PA-1, and organic acids), we successfully developed five different synergetic consortia with improved antimicrobial activity and a broad spectrum of inhibition. These consortia were shown to be effective against a large panel of pathogenic and spoilage microorganisms as well as clinically important multidrug-resistance bacteria. Moreover, because the lower concentrations of bacteriocins and reuterin are used in the synergetic consortia, there is a limited risk of toxicity and resistance development for these compounds.

## INTRODUCTION

The increasing emergence of antibiotic-resistant pathogenic microorganisms is a serious threat to public health across the globe. In agriculture and medicine, the long-term overuse of antibiotics in humans and animals has resulted in extensive bacterial adaptation, leading to them possessing rapidly developed resistances to existing treatments. Moreover, the rates at which bacteria are developing resistances to antibiotics are increasing, while the discovery of new antibiotics remains at a standstill. Therefore, scientists are being encouraged to develop alternative strategies and therapeutic solutions (1).

From a food quality and safety perspective, the use of traditional preservatives such as chemical additives and salt is increasingly being contested in the current context of the “clean label” approach. Thus, consumers are rapidly shifting to natural and minimally processed foods. However, such demands increase the risks associated with foodborne pathogens and spoilage (2). Accordingly, providing safe and high quality food products, without antibiotic residue, and resistance development is challenging for the global food industry; thus, there is an urgent need for alternatives to antibiotics and chemical preservatives.

Scientists in both the clinical and food sectors are under pressure to discover new antimicrobial agents or novel strategies to tackle such problems. In this regard, protective cultures and their antimicrobial compounds have attracted extensive attention as promising alternatives. Protective cultures are antagonistic microorganisms that mainly comprise of lactic acid bacteria, including the genera *Lactobacillus*, *Leuconostoc*, Streptococcus, *Enterococcus*, *and Bacillus* (3). Their bio-protective activities are linked to their ability to metabolically produce different compounds such as organic acids, phenylacetic acid, hydrogen peroxide, diacetyl, reuterin, and bacteriocins (4, 5). Bacteriocins are ribosomally synthesized proteins with high antimicrobial activity in the nanomolar range and have broad or narrow spectrums of inhibition (6). A complete review of bacteriocins and their characteristics is presented in BACTIBASE, a database recently developed by Hammami et al. (7). Bacteriocins are produced by Gram-positive and Gram-negative bacteria and are classified based on the presence or absence of posttranslational modifications in Class I and Class II, which have subclassifications (8). It should be noted that bacteriocins exhibit several characteristics that make them attractive for use in the medical, veterinary, and food sectors, such as only being active against strains that are phylogenetically close to producer strains, their narrower spectrums, strong activity at very low concentrations, the stability to heat, tolerance to extreme salt and pH conditions, and (9) rarer capacities for inducing resistance mechanisms (10). Owing to their potential, approaches that incorporate their application have steadily gained interest in scientific and industrial communities. The potent activity of bacteriocins against significant food spoilage and pathogens in different food matrices, such as meat, vegetables, and dairy products has been well studied (11). As many bacteriocins are naturally produced by lactic acid bacteria in fermented foods, they are generally considered safe. Some have obtained GRAS status by the FDA; including nisin (Nisaplin by Danisco, Chrisin by Chr. Hansen), to control Clostridium botulinum in cheese (12), and meat in particular (13), and colicin E1 for the control of Escherichia coli in beef (14). Kerry Bioscience also markets pediocin PA-1 under the name ALTA 2431 (6). Furthermore, bacteriocins have been shown to be active against significant clinical pathogens such as vancomycin-resistant enterococci (VRE), methicillin-resistant Staphylococcus aureus (MRSA), Clostridium difficile, and Salmonella enterica (15–17). In addition, several bacteriocins are effective in human and animal infection treatments, and some of them have been progressed into clinical evaluation (18).

Despite several studies on the efficacy of bacteriocins as promising antimicrobial agents, the extensive and routine use of these compounds in the food, medical, and veterinary sectors is still limited by several drawbacks such as their narrow spectrum of activity and the development of resistance and cross-resistance between bacteriocins or between antibiotics and bacteriocins.

One of the strategies to overcome these shortcomings is to use a combination of different bacteriocins or bacteriocins with other natural compounds that have different structures and mechanisms of action. Several studies have reported that bacteriocins, in combination with other antimicrobial compounds, effectively inhibit clinical and/or foodborne pathogens (19–22). A synergistic mixture of bacteriocins with other antimicrobials with different mechanisms of action may promote their inhibitory effects and limit the risk of resistance development to either of these compounds. At the same time, a broad spectrum of organisms can be targeted. In an effective synergistic combination of bacteriocins and other antimicrobials, bacteriocins are used in reduced concentrations, which lowers the costs of using these compounds at an industrial scale and makes the approach economically viable (23).

In most reported studies, the selection of bacteriocin combinations (bacteriocin-bacteriocin or bacteriocin-antimicrobials) has been conducted in an arbitrary fashion. In addition, the results obtained are often qualitative and limited to a few bacterial species. A better understanding of the structures and mechanisms of action of the different compounds may allow a better selection of compounds to be tested in combinations and may provide opportunities to identify combinations with additive or synergistic effects. In addition, a more systematic study using a larger panel of food-, medical-, and veterinary-associated pathogenic bacteria will generate more valuable data and more credible scientific evidence regarding the spectrum of inhibition activity obtained by such combinations. This study aimed to investigate the inhibitory activity of various bacteria-derived antimicrobial compounds with different modes of action alone or in combination against primary and secondary panels of pathogenic microorganisms. Nisin Z, pediocin PA-1, microcin J25 (MccJ25), and reuterin were produced and purified. Their antimicrobial activities were evaluated by determining the MIC and minimum bactericidal concentration (MBC) against a first panel of indicator strains, including Gram-positive and Gram-negative bacteria. A broth-based checkerboard assay was carried out to evaluate the interactions (synergetic, additive, or antagonistic) between these compounds: first, two-by-two and then in a combinations of three. Finally, the selected synergetic consortia were tested quantitatively against a secondary panel of pathogenic and spoilage bacteria, including multidrug-resistant bacteria.

## RESULTS

### Production and purification of compounds.

**FIG 1:**
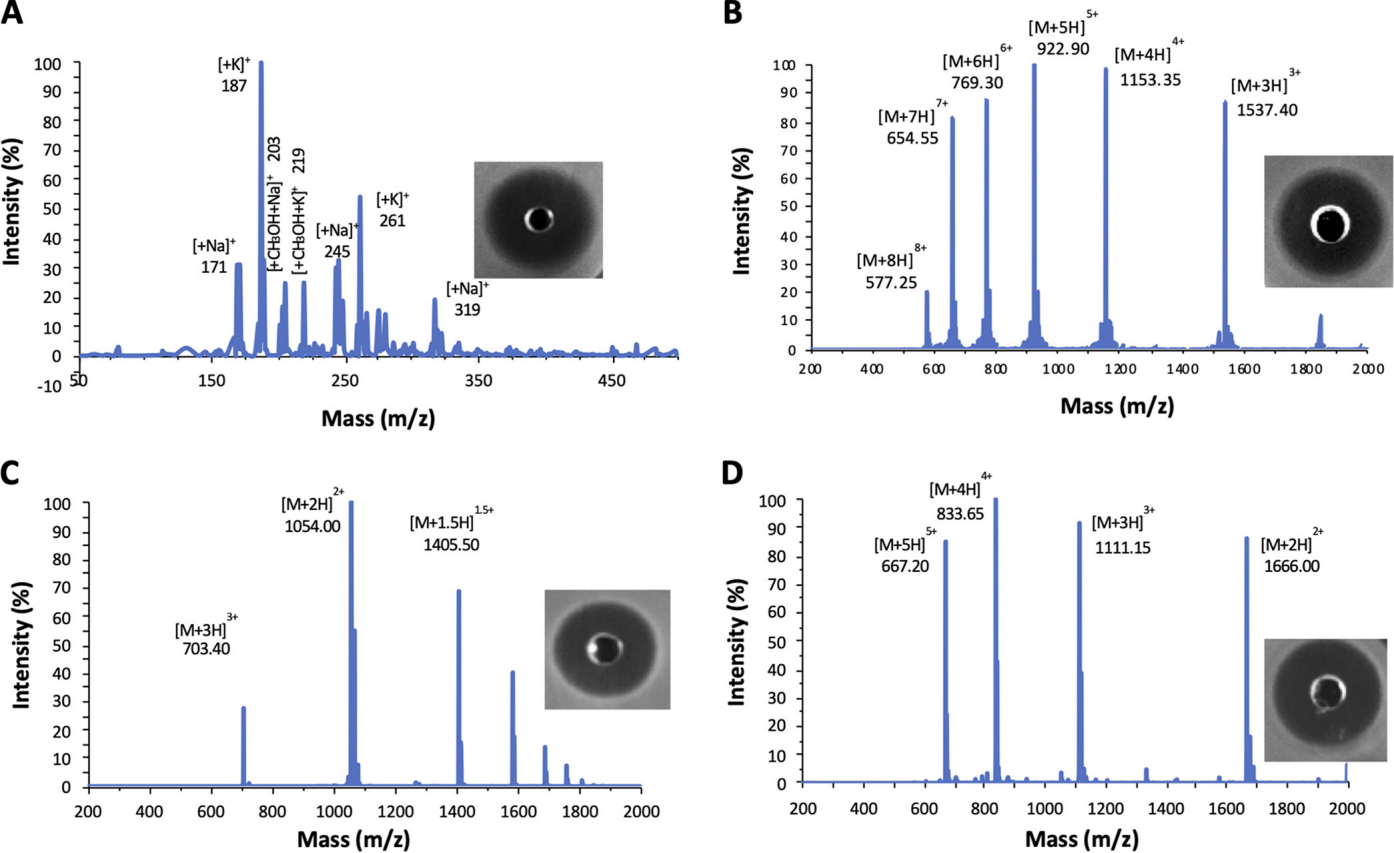
LC/MS and agar well diffusion assay of (A) reuterin, (B) pediocin PA-1, (C) MccJ25, (D) nisin Z.

Reuterin was produced from the bioconversion of glycerol (300 mM) by Lactobacillus reuteri ATCC 53608. We obtained a solution of 200 mM reuterin with a bioconversion yield of 85%, which is active against both indicator strains Listeria ivanovii HPB28 and Salmonella enterica subsp. *enterica* serovar Newport ATCC 6962 (later referred to as *S.* Newport, ATCC 6962) (Fig. 1A). Notably, the reuterin solution did not contain any residual glycerol. Pediocin PA-1 was successfully synthesized with high purity (>90%) and was active against *L. ivanovii* HPB28 (Fig. 1B). Mcc25 was produced by E. coli MC4100 pTUC202, and according to the LC/MS profile, MccJ25 had a purity of >90% after purification (Fig. 1C). Moreover, an activity test showed that MccJ25 was active against *S.* Newport ATCC 6962. Finally, nisin Z was purified from commercial nisin (Niseen^—S^, Fromagex, Canada) with 90% purity (Fig. 1D).

### MIC and MBC of individual compounds.

The MIC and MBC of MccJ25, reuterin, pediocin PA-1, nisin Z, and organic acids against the indicator strains are summarized in Table 1. MccJ25 was the most potent against *S.* Newport ATCC 6962, with a MIC of 0.0356 μg/mL and a MBC value higher than 0.2848 μg/mL. The MBC/MIC ratio of MccJ25 was higher than 8, indicating that MccJ25 exhibited a bacteriostatic mechanism of action. Pediocin PA-1 was the most potent against *L. ivanovii* HPB28, with a MIC of 0.09 μg/mL and no MBC detected (bacteriostatic). The MIC of nisin Z against *L. ivanovii* HPB28 was 1.56 μg/mL, with bactericidal effects observed at a concentration two times that of the MIC (bactericidal at 3.12 μg/mL). As expected, reuterin and acids (citric acid and lactic acid) exhibited a broad spectrum of activity against both Gram-positive and Gram-negative bacteria. Reuterin and lactic acid exhibited slightly lower MICs for *S.* Newport ATCC 6962 (125 μg/mL and 0.2% vol/vol, respectively) than *L. ivanovii* HPB28 (250 μg/mL, and 0.4% vol/vol, respectively). Reuterin at concentrations eight times that of its MI, showed bactericidal effects against both strains. Citric acid presented the same MIC for both strains (0.4% vol/vol). Citric and lactic acid showed bactericidal effects at concentrations four- and 2-fold higher than their MICs, respectively.

**TABLE 1:** The MIC of antimicrobials against *S.* Newport ATCC 6962 and *L. ivanovii* HPB28

Compounds	MIC	MBC concn	MBC/MIC ratio
*S.* Newport ATCC 6962			
MccJ25	0.0356 (μg/mL)	>0.2848 (μg/mL)	>8
Reuterin	125 (μg/mL)	1000 (μg/mL)	8
Citric acid	0.4% (w/v)	1.6% (w/v)	4
Lactic acid	0.2% (v/v)	0.4% (v/v)	2
*L. ivanovii* HPB28			
Pediocin PA-1 PA-1	0.09 (μg/mL)	>0.72 (μg/mL)	>8
Reuterin	250 (μg/mL)	2000 (μg/mL)	8
Nisin	1.56 (μg/mL)	3.12 (μg/mL)	2
Citric acid	0.4% (w/v)	1.6% (w/v)	4
Lactic acid	0.4% (v/v)	0.8% (v/v)	2

### Interactions between antimicrobial compounds (FIC index).

To determine the types of interactions among bacteriocins, organic acids, and reuterin; their fractional inhibitory concentrations were determined based on the MIC values of each individual compound. In general, no antagonistic effects were observed with the different combinations of the antimicrobial compounds tested, while different consortia showed either synergistic or additive effects against Gram-positive or Gram-negative target microorganisms. First, the combinations of the two compounds were evaluated, and their FIC values are listed in Table 2. Nisin Z in combination with either reuterin or pediocin PA-1 showed additive effects against *L. ivanovii* HPB28 (FIC index = 1), and inhibition was obtained at 1/2 MIC nisin Z (0.78 μg/mL) with 1/2 MIC pediocin PA-1 (0.045 μg/mL), or with 1/2 MIC reuterin (125 μg/mL). Similarly, the combination of pediocin PA-1 with reuterin had additive effects (FIC index = 1), and their MICs were reduced to 1/2. The combination of MccJ25 with reuterin was synergistic (FIC index = 0.5), and their MICs were reduced to 1/4 (0.0089 μg/mL and 31.25 μg/mL, respectively) against *S.* Newport ATCC 6962.

**TABLE 2:** FIC values of different combinations of antimicrobials (two compounds) against *L. ivanovii* HPB28 and *S.* Newport ATCC 6962^*a*^

Consortia	Ped-Lac	Ped-Cit	Reu-Cit	Reu-Lac	Ped-Reu	Nis-Reu	Nis-Ped	Nis-Cit	Nis-Lac	Cit-Lac
*L. ivanovii* HPB28										
FIC	0.5-0.75	0.5-0.75	0.75	0.75	1	1	1	0.625	0.75	0.75
MIC	0.045-0.1	0.045-0.1	125-0.1	125-0.1	0.045−125	0.78−125	0.78−0.045	0.78-0.05	0.78-0.1	0.2-0.1
*S.* Newport ATCC 6962										
		J25 -Reu	J25 -Lac		J25 -Cit	Reu-Cit		Reu-Lac	Cit-Lac	
FIC		0.5	0.5		0.5	0.5		0.5	0.75	
MIC		0.0089-31.25	0.0089-0.05		0.0089-0.1	31.25-0.1		31.25-0.05	0.2-0.05	

a J25: MccJ25, Cit: citric acid, Lac: lactic acid, Reu: reuterin, Ped: pediocin PA-1, Nis: nisin.

When the selected bacteriocins and reuterin were combined with citric acid or lactic acid, synergistic to partial synergistic activity against the indicator strains was noted. Nisin Z in combination with citric acid (FIC index = 0.625) or with lactic acid (FIC index = 0.75), exerted a partial synergistic effect against *L. ivanovii* HPB28, and its MIC in combinations was reduced to 1/2 (0.78 μg/mL). When MccJ25 was combined with citric acid or lactic acid, we observed a synergistic interaction (FIC index = 0.5), and their MICs against *S.* Newport ATCC 6962 were reduced to 1/4 (0.0089 μg/mL MccJ25 and 0.1% wt/vol of citric acid or 0.05% lactic acid). Moreover, the combination of reuterin with citric acid or lactic acid showed synergistic activity (FIC index = 0.5), and inhibition was obtained at lower concentrations, corresponding to 1/4 MICs (31.25 μg/mL reuterin and 0.05% vol/vol lactic acid or 0.1% wt/vol citric acid) against *S.* Newport ATCC 6962. However, the same combination against *L. ivanovii* HPB28 showed a partial synergistic effect (FIC index = 0.75) and inhibition at 1/2 and 1/4 of their MICs, respectively (125 μg/mL reuterin and 0.1% wt/vol citric acid or 0.1% vol/vol lactic acid). Pediocin PA-1 combined with either citric acid or lactic acid acted synergistically to partially synergistically and could inhibit *L. ivanovii* HPB28 at 1/2 - 1/4 of their MICs (FIC index = 0.5 -0.75).

After evaluating the interactions between each pair of compounds, the combinations of three were evaluated based on their interactions. For this purpose, the combinations of each of the two compounds previously described were now considered one at their reduced MICs, and their interactions with the third compound were determined. The FIC values are listed in Table 3. MccJ25 with reuterin + lactic acid exerted a synergistic interaction (FIC index = 0.5); the MICs for MccJ25 reduced to 1/4 (0.0089 μg/mL) and those for reuterin and lactic acid reduced to 1/16 (7.8 μg/mL and lactic acid 0.0125% wt/vol, respectively). Similar results were observed with the combination of MccJ25 and reuterin + citric acid (FIC index = 0.5) against *S.* Newport ATCC 6962. The combination of MccJ25 with citric acid + lactic acid showed a synergistic effect (FIC index = 0.5), and inhibition was obtained at 1/4 MIC MccJ25 (0.0089 μg/mL), 1/8 MIC citric acid (0.05% wt/vol), and 1/16 MIC lactic acid (0.0125%). Similarly, when reuterin was combined with citric + lactic acid, a synergistic interaction against *S.* Newport ATCC 6962 was apparent (FIC index = 0.5, 1/4 MIC reuterin), and they showed a partially synergistic activity (FIC index = 0.75, 1/2 MIC reuterin) against *L. ivanovii* HPB28. Similarly, the combination of pediocin PA-1 with two acids was partially synergistic (FIC index = 0.75), and their MICs were reduced to 1/2 MIC of pediocin PA-1 (0.045 μg/mL), 1/8 MIC citric acid (0.05% wt/vol), and 1/16 MIC lactic acid (0.025% vol/vol).

**TABLE 3:** FIC values of different combinations of antimicrobials (three compounds) against *S.* Newport ATCC 6962 and *L. ivanovii* HPB28

Consortia	MccJ25 + (reuterin + lactic)	MccJ25 + (reuterin + citric)	MccJ25 + (citric + lactic)	Reuterin + (citric + lactic)
*S.* Newport ATCC 6962				
FIC value	0.5	0.5	0.5	0.5
MIC	0.0089 + (7.8 + 0.0125)	0.0089 + (7.8 + 0.025)	0.0089 + (0.05 + 0.0125)	31.25 + (0.05 + 0.0125)
*L. ivanovii* HPB28				
Consortia		Pediocin PA−1 + (citric + lactic)	Reuterin + (citric + lactic)	
FIC value		0.75	0.75	
MIC		0.045 + (0.05 + 0.025)	125 + (0.05 + 0.025)	

**FIG 2:**
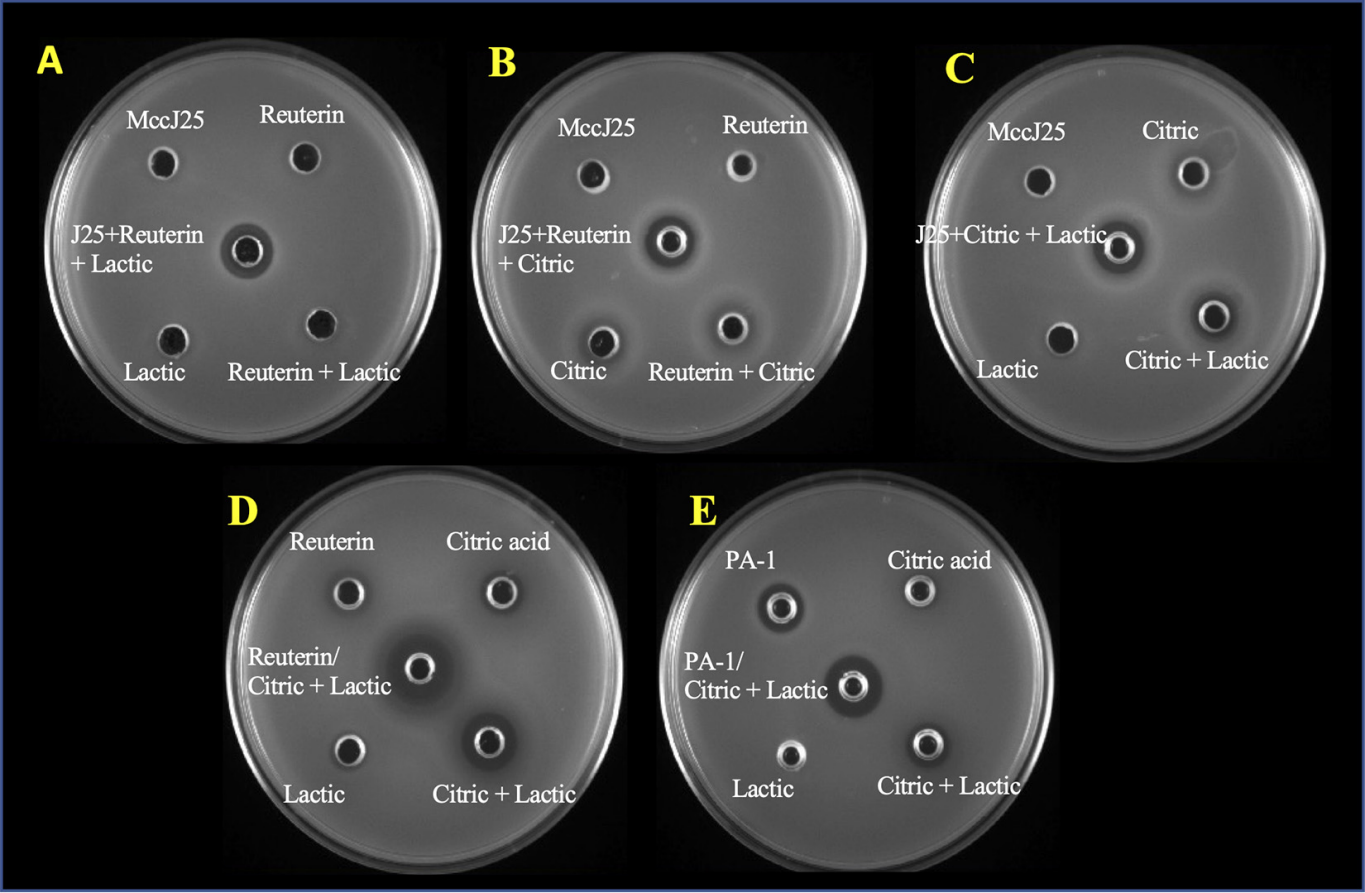
Agar well diffusion assay depicting inhibitory effect of compounds alone and in combinations A: MccJ25 + (reuterin + lactic) acid against *S.* Newport ATCC 6962. B: MccJ25 + (reuterin + citric acid) against *S.* Newport ATCC 6962. C: MccJ25 + (citric acid + lactic acid) against *S.* Newport ATCC 6962. D: Reuterin + (citric + lactic acid) against *S.* Newport ATCC 6962. E: Pediocin PA-1 + (citric acid + lactic acid) against *L. ivanovii* HPB28. All samples are tested at 50 times MICs given in Table 3.

**(i) Analyses of synergetic consortia with agar diffusion assay.** Five combinations containing three different compounds were selected for their synergetic effects. They were further qualitatively analyzed using the agar well diffusion assay (Fig. 2). To obtain distinct inhibition zones, all compounds were tested at 50 times the MICs given in Table 3. The synergistic combination containing MccJ25 and reuterin + lactic acid (Fig. 2A) produced a clear 13 mm inhibition zone against *S.* Newport ATCC 6962; whereas no inhibition was observed when each compound was tested alone at the same concentrations used in the consortium. Similarly, MccJ25, in combination with reuterin + citric acid (Fig. 2B), produced a 13 mm inhibition zone. The combination of MccJ25 with citric + lactic acid also produced an inhibition zone of 13 mm (Fig. 2C); however, there was no inhibition zone for each individual compound at the same concentrations, and only citric + lactic acid gave an inhibition zone, that was 11 mm. As shown in Fig. 2D, the diameter of the inhibition zone against *S.* Newport ATCC 6962 increased when reuterin was combined with citric + lactic acid (16 mm), relative to that induced by the individual compounds at the same concentrations (for reuterin or citric acid: 10 mm, and for citric + lactic acid: 13 mm). Figure 2E shows that the combination of pediocin PA-1 with citric and lactic acid resulted in a larger zone of inhibition against *L. ivanovii* HPB28 (14 mm) compared with individual compounds (10 mm for citric + lactic acid, and 11 mm for pediocin PA-1).

**FIG 3:**
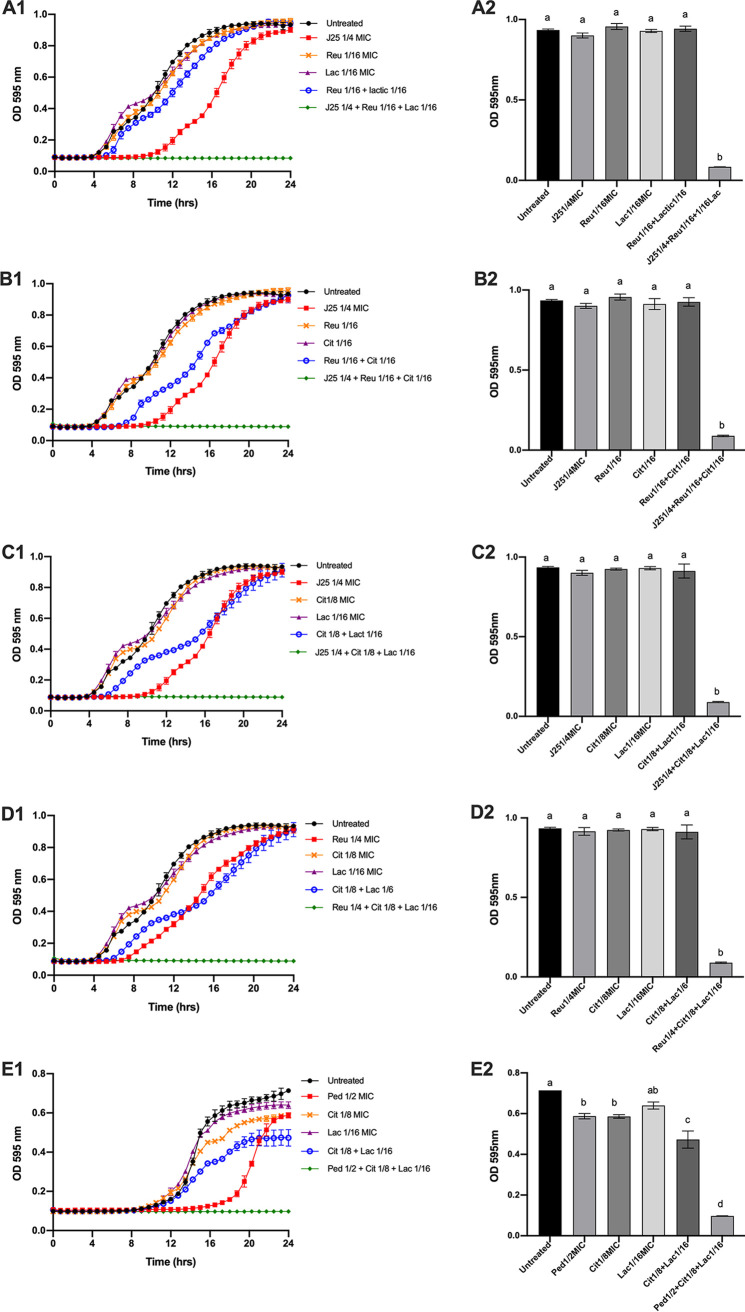
Growth curves and bar charts (OD_595_ at 24 h) analysis of (A1, A2) *S. newport* ATCC 6962 in the presence of MccJ25 1/4 MIC, reuterin 1/16 MIC, lactic 1/16 MIC, and combinations thereof (B1, B2) *S. newport* ATCC 6962 in the presence of MccJ25 1/4 MIC, reuterin 1/16 MIC, citric 1/16 MIC, and combinations thereof (C1, C2) *S. newport* ATCC 6962 in the presence of MccJ25 1/4 MIC, citric 1/8 MIC, lactic 1/16 MIC, and combinations thereof (D1, D2) *S. newport* ATCC 6962 in the presence of reuterin 1/4 MIC, citric 1/8 MIC, lactic 1/16 MIC, and combinations thereof (E1, E2) *L. ivanovii HPB28* in the presence of pediocin PA-1 1/4 MIC, citric 1/8 MIC, lactic 1/16 MIC, and combination thereof. The experiment was performed in triplicates and the error bars indicate the standard deviation of the mean. Treatment with different alphabetic letters is statistically different (Tukey’s *t* test) at *P < *0.001. J25: MccJ25, Cit: citric acid, Lac: lactic acid, Reu: reuterin, Ped: pediocin PA-1.

**(ii) Impacts of the antimicrobial compounds on growth kinetics.** The inhibitory activities of the five combinations (of three compounds) with synergistic effects were studied over an incubation period of 24 h. As shown in Fig. 3, all synergistic consortia could completely inhibited the growth of the indicator strains. When MccJ25 + reuterin + lactic acid (1/4 MIC, 1/16 MIC, and 1/16 MIC), MccJ25 + reuterin + citric acid (1/4 MIC, 1/16 MIC, and 1/16 MIC), and MccJ25 + citric + lactic acid (1/4 MIC, 1/8 MIC, and 1/16 MIC) were used against *S.* Newport ATCC 6962, complete inhibition of growth was observed over a 24 h period (Fig. 3A1-C1). However, except MccJ25, for which a 5 h delay in the growth (extended lag phase) was observed, each of the antimicrobials did not show inhibition when independently applied. For 1/8 MIC citric acid + 1/16 MIC lactic acid and 1/16 MIC reuterin + 1/16 MIC citric acid, a time lag of approximately 2–3 h was noted, compared with the control. The combination of 1/4 MIC reuterin + 1/8 MIC citric + 1/16 MIC lactic acid was sufficient to completely inhibit the growth of *S.* Newport ATCC 6962, while none of the individual compounds showed any inhibitory effects (Fig. 3D1). A combination of 1/2 MIC pediocin PA-1 + 1/8 MIC citric + 1/16 MIC lactic acid resulted in the complete inhibition of *L. ivanovii* HPB28 (Fig. 3E1). Antimicrobials alone did not show any inhibitory effects over 24 h, and only pediocin PA-1 at 1/2 MIC delayed the growth for about 6 h. Altogether, these results provide further evidence of the synergy and antimicrobial efficacy observed when two or three compounds were used in combinations. We have also compared OD_595_ at 24 h using on-way analysis of variance (ANOVA) with Tukey’s *t* test. In general, the bacterial growth in the presence of different consortia at 24 h were statistically different from the growth obtained in the presence of each compound alone (*P < *0.001) (Fig. 3A2, B2, C2, D2, E2).

### Antimicrobial activity against a secondary panel of pathogen, spoilage and multidrug-resistant bacteria.

One of the objectives of combining several antimicrobial compounds is to extend the spectrum of antimicrobial activity. The five selected synergistic combinations were tested against a secondary panel of pathogenic and spoilage organisms (Table 4). Most of the strains were inhibited by the selected consortia. Notably, developed consortia showed synergistic effect against most of strains tested (Table 4). Interestingly, when antimicrobial compounds were individually tested, against the same secondary panel, at the same concentrations used in the synergistic consortia, no significant inhibitory effects were observed, and their MIC values were significantly higher (Table S1 Supporting Information).

**TABLE 4:** FIC index values of five developed consortia against panel of spoilage and pathogenic strains^*a*^

	FIC index				
Bacteria strain	Reuterin+citric+lactic	MccJ25+reuterin+citric	MccJ25+reuterin+lactic	MccJ25+citric+lactic	Pediocin+citric+lactic
Listeria monocytogenes ATCC 19112	FIC = 0.375	—^*b*^	—	—	FIC = 0.5
Staphylococcus aureus ATCC 6538	FIC = 0.5	—	—	—	FIC =1
Bacillus cereus ATCC 14579	FIC = 0.25	—	—	—	FIC = 0.5
Enterococcus faecalis ATCC 29212	FIC = 0.375	—	—	—	FIC = 0.5
Listeria innocua ATCC 51742	FIC = 0.25	—	—	—	FIC = 0.625
Brochothrix thermosphacta ATCC 11509	FIC = 0.625	—	—	—	FIC = 1
Lactobacillus acidophilus ATCC 4356	FIC = 1	—	—	—	FIC > 1
*Lactococcus cremoris* ATCC 19257	FIC >1	—	—	—	FIC > 1
Lactobacillus casei ATCC 334	FIC = 0.375	—	—	—	FIC = 0.625
Pediococcus pentosaceus ATCC 33316	FIC = 1	—	—	—	FIC = 1
*Carnobacterium divergence* ATCC 35677	FIC = 0.375	—	—	—	FIC = 0.625
Pseudomonas aeruginosa ATCC 15442	FIC = 0.25	FIC = 0.375	FIC = 0.5	FIC = 0.375	—
Escherichia coli ATCC 35150	FIC = 0.5	FIC = 0.5	FIC = 0.5	FIC = 0.375	—
Aeromonas hydrophila ATCC 7966	FIC = 0.5	FIC = 0.75	FIC = 0.75	FIC = 0.75	—
Klebsiella *pneumonae* *ATCC 13883*	FIC = 0.375	FIC = 0.5	FIC = 0.375	FIC > 1	—
Campylobacter coli 2020/0011	FIC = 0.25	FIC = 0.375	FIC = 0.375	FIC = 0.375	—
Enterobacter aerogenes ATCC 13048	FIC = 0.25	FIC = 0.75	FIC = 0.75	0FIC 0.5	—
Salmonella *enteria Minnesota* ATCC 9700	FIC = 0.5	FIC = 0.625	FIC = 0.625	FIC = 0.5	—
Salmonella enterica *Typhimurium* ATCC 14028	FIC = 0.375	FIC = 0.5	FIC = 0.5	FIC = 0.375	—

a Reuterin + citric + lactic acid, MccJ25 + reuterin + citric acid, MccJ25 + reuterin + lactic acid, MccJ25 + citric acid + lactic acid, pediocin PA-1 + citric acid + lactic acid.

b —, Refers to no inhibition was observed at tested concentrations.

Synergistic consortia were also tested for their inhibitory activity against five multiresistant strains, namely, S. aureus 40709611, Streptococcus uberis 30600126, E. coli C999, S. enterica Enteritidis C664, and Klebsiella pneumoniae C1865. As shown in Table 5, the use of antimicrobials alone resulted in either no activity or activity at high MICs. However, when the same compounds were used in combination, all five multiresistant strains were successfully inhibited (Table 6). All consortia showed synergistic effect against these strains with exception of pediocin PA-1 + citric acid + lactic acids.

**TABLE 5:** The MIC of antimicrobials against multiresistant strains

		Minimum inhibitory concn				
Bacterial strain	Resistant profile	Citric acid %w/v	Lactic acid %v/v	Reuterin μg/mL	MccJ25 μg/mL	Pediocin PA-1 μg/mL
S. aureus 40709611	PEN-CF-CTX-FOX-CIP-CC-KAN-GEN-STR-P/N	0.312	0.156	100	—^*a*^	>250
*S. uberis* 30600126	PEN-AMO-VAN-CEP-CTX-FOX-ERY-CC-ERY-P/N	0.156	0.312	200	—	>250
E. coli C999	CTX-AMP-STR-KAN-TET-TOB-SUL SXT-NAL-CIP	1.25	0.625	400	283	—
S. enterica *Enteritidis* C664	CTX-AMP-STR-TET-SUL-SXT-NAL	0.625	0.312	200	141.8	—
*K. pneumonia* C1865	CTX, AMP, TOB, ATM, KAN, TET, CHL, SXT, SUL, CIP, NAL, NOR	0.625	0.312	200	141.8	—

a —, Refers to no inhibition was observed at tested concentrations.

**TABLE 6:** FIC index values of five developed consortia against multi-resistant strains^*a*^

	FIC index				
Bacterial strain	Reuterin-Citric-Lactic	MccJ25/reuterin/citric	MccJ25/reuterin/lactic	MccJ25/citric/lactic	Pediocin /citric/lactic
S. aureus 40709611	FIC = 0.25	—^*b*^	—	—	FIC > 1
*S. uberis* 30600126	FI = 0.5	—	—	—	FIC = 1
E. coli C999	FIC = 0.25	FIC = 0.375	FIC = 0.375	0.375	—
S. enterica *Enteritidis* C664	FIC = 0.25	FIC = 0.625	FIC = 0.625	FIC = 0.75	—
*K. pneumonia* C1865	FIC = 0.25	FIC = 0.625	FIC = 0.625	FIC = 0.75	—

a Reuterin + citric + lactic acid, MccJ25 + reuterin + citric acid, MccJ25 + reuterin + lactic acid, MccJ25 + citric acid + lactic acid, pediocin PA-1 + citric acid + lactic acid.

b —, Refers to no inhibition was observed at tested concentrations.

## DISCUSSION

Natural antimicrobial agents, including bacteriocins, have attracted extensive attention as a new microbial barrier in both the food and veterinary sectors. In the food sector, bacteriocins are an ideal option for improving food quality and safety, as a replacement for controversial chemical preservatives (24). In the veterinary and medical sectors, where the search for novel antimicrobials has become an urgent task, bacteriocins are very attractive alternatives to antibiotics, for the prevention and treatment of bacterial infections (8, 25). Because of their narrow spectrums of inhibition, using a bacteriocin alone will considerably limit its activity and increase the risk of developing resistant variants. Combining different bacteriocins or natural antimicrobial agents (stressors) may become a particularly appealing approach, as the synergistic activity of antimicrobials may reduce the development of resistances in pathogenic bacteria, while a broad spectrum of pathogens or spoilage organisms can be targeted at lower dosages (26). Some studies have demonstrated synergistic effects between bacteriocins and antibiotics against important clinical pathogens (27–29). However, very few studies have investigated the synergistic effects of multiple bacteriocins that belong to different classes or that have different mechanisms of action. Moreover, the interactions between bacteriocins and other natural antimicrobial compounds such as organic acids and reuterin have not been thoroughly investigated. In this study, our approach was to combine natural antimicrobial compounds with different structures and modes of action. Such an approach would improve their inhibitory activity, broaden their spectrum of antimicrobial activity and possibly limit the development of bacterial resistances to these compounds.

This study investigated the synergistic interactions between different bacteriocins, and organic acids, and/or reuterin. Previous studies have addressed the antimicrobial activity of bacteriocins in combination with other stressors such as organic acids (30), other bacteriocins (31), essential oils (32, 33), cinnamon (34), and EDTA (35). However, these combinations are often designed arbitrarily and do not consider the specificities of the different compounds and, more particularly, their structure, the differences in their mechanisms of action, or their spectra of activity. More targeted combinations were designed and tested in this study with the ultimate goal of ensuring a more potent and broader spectrum of inhibition.

Pediocin PA-1, a class IIa bacteriocin, has a very narrow spectrum of inhibition directed mainly against the clinically relevant and foodborne pathogen, *Listeria* spp. However, pediocin PA-1 is not active against many other Gram-positive and Gram-negative bacteria. To enhance its antimicrobial activity, pediocin PA-1 was combined with other antimicrobials, such as organic acids and other bacteriocins. In this study, the combined application of pediocin PA-1 and nisin Z demonstrated an additive effect against *L. ivanovii* HPB28, which is in agreement with the findings of another study (36). Moreover, our results indicated that pediocin PA-1 with citric acid and/or lactic acid exhibited synergistic to partially synergistic interactions (the MIC of pediocin PA-1 reduced to 1/2 or 1/4 MICs when combined with citric acid or lactic acid) against *L. ivanovii* HPB28. Furthermore, Gram-negative strains such as Aeromonas hydrophila *and* Klebsiella pneumoniae could be inhibited by this combination. In this consortium, organic acids likely initially act as permeabilizing agents, allowing pediocin PA-1 to access its target receptor and the cytoplasm. Notably, a recent study has reported that the combination of pediocin PA-1 with lactic acid could synergistically inhibit the Gram-negative A. hydrophila, which is in line with our findings (37). Lactic acid releases the outer membrane LPS, granting pediocin PA-1 access to the cytoplasmic membrane, causing cell death by the dissipation of the proton motive force in the inner membrane (38).

Reuterin is an aldehyde with a broad inhibitory effect and great potential for applications in food and clinical settings. Recently, we have shown that reuterin may exhibit some toxic effects on epithelial cells and may cause hemolysis at concentrations above 20 mg/mL (39). Combining reuterin with other antimicrobial compounds reduces the MIC, thereby reducing the risks of developing side effects. Very few studies have reported on the combination of reuterin and other antimicrobial agents. In this study, the combination of reuterin with either bacteriocins, citric acid, or lactic acid against Gram-positive and Gram-negative indicator strains was synergistic to partially synergistic. Although there is a scarcity of studies addressing the combination of reuterin with bacteriocins, few studies have reported the enhanced activity of reuterin with nisin Z (40) and durancin 61A (19). In this study, reuterin in combination with nisin Z showed additive effects against *L. ivanovii* HPB28. This result was in line with a previous study that reported on the enhanced activity of reuterin, when combined with nisin Z, against Listeria monocytogenes (41). Similarly, we found that the combination of reuterin with pediocin PA-1 exerted an additive effect against *L. ivanovii* HPB28, owing to the multitargeting mechanisms (8, 42).

Notably, no antagonistic interactions were observed for any of the different combinations of antimicrobial compounds evaluated. This result reflects the advantages of selecting antimicrobials with different mechanisms of action to develop combinations with additive or synergistic effects.

To the best of our knowledge, Gram-negative bacteriocins have not been investigated for any potential synergistic combinations. MccJ25 is one of the most well-known Gram-negative bacteriocins produced by E. coli. In this study, we determined the synergistic interactions between MccJ25 and two different organic acids (citric and lactic acid), which could be due to the membrane permeabilization action of organic acids and subsequent access that MccJ25 has to the cytoplasm. Moreover, the combination of MccJ25 and reuterin, exhibited synergistic activity at very low concentrations (1/4 MIC for each). MccJ25 showed dual independent mechanisms of action; one was the inhibition of vital bacterial enzymatic function DNA-dependent RNA polymerase (43). Reuterin exerts its effect by reacting with thiol groups and causing oxidation, followed by cell membrane disruption, DNA damage, and consequently, cell death (42, 44). Their different modes of action might be responsible for their synergistic interaction; however, further in-depth studies are required.

Enhancing the activity of bacteriocins to target a broader spectrum of antimicrobial activity is of paramount importance for the food industry, as it allows better control of pathogenic and spoilage microorganisms and ensures a longer shelf life for foods. This is of particular importance in the current context, where the use of chemical additives is controversial and where consumers’ demand for more natural products is constantly increasing. One of the most important features for the use of antimicrobial combinations is the possibility of simultaneously targeting Gram-positive and Gram-negative pathogenic and spoilage bacteria. Here, we investigated the efficacy of the developed consortia against a broad panel of spoilage and pathogenic bacterial strains. Most of the strains tested were successfully inhibited by all consortia, but reuterin and organic acids showed the greatest synergistic effects against all strains. This result could be due to the broad spectrum of inhibition of both reuterin and organic acids and their complementary mechanisms of action against both Gram-positive and Gram-negative bacteria.

Considering the potential use of bacteriocins and reuterin in clinical settings, we investigated the efficacy of the developed consortia against five multidrug-resistant pathogenic strains. All of the developed consortia were effective against the tested strains, whereas the combination of reuterin with acids exerted high potency with strong synergistic activity against all multiresistant strains. Similarly, Hanchi and coworkers reported on the synergistic inhibition of the reuterin and durancin 61A combination against *C. difficiIe* which was attributed to their different modes of action (19). Taken together, these results show that consortia, particularly the reuterin combinations, could be considered for different applications in clinical settings.

### Conclusion.

Our study demonstrated that different combinations of bacteriocins, reuterin, and organic acids showed synergistic inhibitory effects. The selection process based on different mechanisms of action resulted in novel antimicrobial combinations with high potency, that may target a broad spectrum of bacteria, and possibly reduce the risks of resistance development. Our study also showed that these synergetic consortia were active against antibiotic-resistant pathogens. The use of a low concentrations of bacteriocins and reuterin in the synergetic consortia is both beneficial from a toxicity perspective and economical for industrial applications. More in-depth studies should be performed to prove the efficacy of these combinations under actual usage conditions.

## MATERIALS AND METHODS

### Strains and growth condition.

*L. ivanovii* HPB28 (Public Health Agency of Canada) and *S.* Newport ATCC 6962 (STELA Collection, Laval University) were used as indicator strains. *L. ivanovii* HPB28 culture was prepared by inoculating 10 mL of Tryptone Soy Broth TSB enriched with 0.6% yeast extract with a single colony from TSB agar (1.5%, Oxide) plate grown overnight at 30°C. *S.* Newport ATCC 6962 culture was prepared by inoculating 10 mL of LB with a single colony from LB agar plate grown overnight at 37°C.

Lactobacillus reuteri ATCC 53608, used for reuterin production, was cultured at 37°C overnight in De Man Rogosa Sharpe (MRS) (Oxoid, Nepean, ON, Canada), under anaerobic condition (10% H_2_, 10% CO_2_, 80% N_2_) in Forma Anaerobic Chamber (Thermo Scientific, Waltman, MA, USA).

E. coli MC4100 PTUC 202 (STELA Collection, Laval University), used for MccJ25 production, was cultured aerobically at 37°C overnight in Luria-Bertani (LB) (Difco Laboratories, Spark, MD, USA). All bacterial stocks were stored at −80°C in their respective media, supplemented with 20% sterile glycerol.

The strains used as a secondary panel for screening selected consortia are listed in Table S2 of (supplemental materials) and were acquired from the Laval University culture collection (Laval University, Canada).

### Production of antimicrobial compounds.

**(i) Reuterin.** It was produced by L. reuteri using the following protocol: Culture of L. reuteri was grown anaerobically in MRS media supplemented with 20 mM glycerol at 37°C overnight. After incubation, the culture was centrifuged (1500 × *g*, 10 min, 20°C), the cells were washed twice (with 0.1 M potassium phosphate buffer pH 7) to be resuspended in 300 mM glycerol and incubated anaerobically at room temperature for 45 min. Bacterial suspensions were centrifuged (10,000 × *g*, 10 min, 4°C), and the supernatant was filtered and lyophilized. Finally, the purity and quantity of reuterin were verified using an analytical HPLC system (Waters, Milford, MA) equipped with ICsep-ion-300 column (Transgenomic, San Jose, CA) as previously described (39).

**(ii) Microcin J25.** It was obtained from the culture supernatant of E. coli MC400 PTUC202 cultured in minimal medium (M63) using previously established conditions (45). MccJ25 was purified from the culture supernatant by solid-phase extraction using a Sep-Pak C18 35 cc Cartridge (Water). Mcc J25 was eluted by acetonitrile/water (30% vol/vol) containing 0.1% HCl and further purified to homogeneity (up to 95% purity) by RP-HPLC (Beckman Coulter System Gold Preparative HPLC system, Mississauga, ON, Canada) on a preparative C18 column (Luna 10 μm, 250 mm x 21.10 mm, Phenomenex, CA, USA) at a flow rate of 10 mL/min. The purified sample was quantified by RP-HPLC system (Waters, Milford, MA) equipped with an analytical C18 column (Aeris 3.6 μm PEPTIDE XB-C18, 250 × 4.6 mm, Phenomenex, CA USA) according to (45).

**(iii) Pediocin PA-1.** It was prepared by standard solid-phase peptide synthesis (SPSS) according to (46). Pediocin PA-1 was purified to over 95% homogeneity using RP-HPLC, and mass spectrometry analysis was performed to confirm identity and purity before use.

**(iv) Nisin Z.** It was isolated from a commercial nisin solution purchased from Niseen^—S^, Fromagex, Canada. The commercial solution was purified by Sep-Pack C18 column 35 cc Cartridge (Water) with a flow rate of 2.5 mL/min. And samples containing nisin Z were concentrated by Speed-Vac overnight at 45°C.

Citric acid and lactic acid were purchased from Sigma-Aldrich.

### LC/MS-MS analysis.

The purity of the samples was determined by LC/MS-MS on a Waters Synapt G2-SI with a Waters UPLC binary pump. The mass spectrometer was performed in high-resolution mode, and calibration was done with sodium formate (Sigma) solution and lock-mass correction using a leucine-enkephaline solution (Waters).

### Agar well diffusion assay.

Inhibitory activity was determined qualitatively by agar well diffusion assay as described previously (45). *L. ivanovii* HPB28 and *S.* Newport ATCC 6962 were seeded at 1% in the appropriate media (soft agar). In agar plates, a 5 mm well was created by sterile pipet glass. 80 μL of samples were added and incubated under the required condition.

### MIC and MBC assay.

MIC and MBC of antimicrobial compounds were determined according to clinic and Laboratory Standard Institution (CLSI) guidelines (47). In brief, stock solutions (MccJ25 1 μg/mL, pediocin PA-1 12 μg/mL, reuterin 15 μg/mL, citric acid 50% wt/vol, lactic acid 50% vol/vol) were prepared for each antimicrobial compound using sterile distilled water. In a sterile flat-bottom 96-well polystyrene microtiter plate, a 2-fold serial dilution of each compound (125 μL) was performed in 125 μL of appropriate medium. Target strains were subcultured from an overnight grown culture and allowed to grow to an optical density OD_595_ of ∼ 0.5, diluted in an appropriate medium to a final concentration of 10^5^ CFU/mL and 50 μL was added to each well. After incubation, optical densities at 595 nm (OD_595_) were measured (Infinite M200, Tecan, Switzerland), and MIC was noted as the lowest concentration of antimicrobials with less than 90% growth compared to control based on optical density measurement. Growth curves were obtained spectrophotometrically using (Infinite M200, Tecan, Switzerland); OD_595_ was measured at a different time interval for 24 h.

For MBCs determination, 10 μl was withdrawn from each clear well, showing complete inhibition of the tested strain, plated on agar medium and incubated under the same condition as the previous test. MBCs were determined as the lowest concentration from which the number of colonies on a subculture plate was less than 0.1% of the initial inoculum (indicating 99.9% or more killing and bactericidal effect has been achieved).

### Checkerboard/FIC assay.

For the FIC index experiment, a combination of two different antimicrobials (e.g., A and B) was determined, and then the combination of three was determined. On the basis of the MIC value of each antimicrobial compound against a specific strain, a 2-fold serial dilution of compound A was made horizontally in broth medium in a 96-well microtiter plate, starting at a concentration of 32 times the MIC. For compound B, a similar serial dilution was prepared vertically starting at eight times the MIC, and 50 μL of compound B was transferred to the original microplate containing a solution of compound A. Bacterial strain was added as described in the MIC assay, and plates were incubated under acquired conditions. The FIC index was calculated by the following equation: FIC = FIC A + FIC B = (A/MIC_A_) + (B/MIC_B_). A is the minimum concentration of antimicrobial A used in combination with another to achieve the antimicrobial effect, and MIC_A_ is the MIC of compound A alone against target strain. FIC index data were interpreted as follows: FIC ≤ 0.5 is synergy, 0.5 < FIC ≤ 0.75 is partially synergy, 0.75 < FIC ≤ 1 is additive, and FIC > 1 is indifferent and FIC > 4 is antagonistic. All compounds were initially analyzed in two combinations. Depending on their FIC concentrations, three combinations were analyzed where A and B were considered in a mixture as one compound, and their FIC concentration was considered their new MICs together. They were analyzed with compound C as three combinations.

### Statistical analysis.

All the experiments were carried in triplicate, and data were analyzed using a data analysis tool in Excel 365 (Microsoft, WA, USA). Statistical analyses were performed by on-way analysis of variance (ANOVA) with Tukey’s *t* test using GraphPad Prism 8 (GraphPad Software, San Diego, CA, USA). Also, growth curves were fitted using GraphPad Prism 8.
