# Supplementary material for: Bacteriocin-Based Synergetic Consortia: a Promising Strategy to Enhance Antimicrobial Activity and Broaden the Spectrum of Inhibition
Source: Microbiol Spectr. 2022 Feb 16;10(1):e00406-21. doi: 10.1128/spectrum.00406-21 (PMC8849083; doi:10.1128/spectrum.00406-21)
Supplement: SUPPLEMENTAL FILE 1 — Supplemental material. Download SPECTRUM00406-21_Supp_1_seq1.pdf, PDF file, 0.2 MB [file spectrum00406-21_supp_1_seq1.pdf]

Table S1. minimum inhibitory concentration for a secondary panel of strains

Bacterial strain	Minimum Inhibitory Concentration				
	**Citric acid %w/v**	**Lactic acid %v/v**	**Reuterin mg/mL**	**MccJ25 µg/mL**	**Pediocin PA-1 µg/mL**
Listeria monocytogenes ATCC 19112	0.625	0.312	400	--	0.625
Staphylococcus aureus ATCC 6538	0.156	0.312	200	--	>250
Bacillus cereus ATCC 14579	0.625	0.625	200	--	>250
Enterococcus faecalis ATCC 29212	0.625	0.625	800	--	12.5
Listeria innocua ATCC 51742	0.625	0.625	800	--	1.25
Brochothrix thermosphacta ATCC 11509	0.312	0.312	400	--	>250
Lactobacillus acidophilus ATCC 4356	0.312	0.625	200	--	>250
Lactococcus cremoris ATCC 19257	0.312	0.312	400	--	>250
Lactobacillus casei ATCC 334	1.25	0.625	400	--	>250
Pediococcus pentosaceus ATCC33316	1.25	0.625	400	--	12.5
Carnobacterium divergence ATCC 35677	0.625	0.312	800	--	0.78
Pseudomonas aeruginosa ATCC 15442	0.625	0.625	200	284	--
Escherichia Coli ATCC 35150	1.25	0.625	200	1142	--
Aeromonas hydrophila ATCC 7966	0.039	0.039	50	28.4	--
Klebsiella pneumonae ATCC13883	0.078	0.078	50	56.95	--
Campylobacter coli 2020/0011	0.156	0.156	12.9	35.59	--
Enterobacter aerogenes ATCC 13048	0.312	0.156	100	142	--
Salmonella enteria Minnesota ATCC 9700	0.156	0.156	50	56.95	--
Salmonella enterica Typhimurium ATCC 14028	0.156	0.156	100	113.9	--

Table S2. Bacterial strains and culture condition in this work

Organisms	Culture media	Culture condition[1]
Listeria monocytogenes ATCC 19112	BHI	37°C, aerobic
Staphylococcus aureus ATCC 6538	TSB	37°C, aerobic
Clostridium perfringens	RCM	37°C, anaerobic
Bacillus cereus ATCC 14579	TSB	30°C, aerobic
Enterococcus faecalis ATCC 29212	BHI	37°C, aerobic
Listeria innocua ATCC 51742	BHI	37°C, aerobic
Brochothrix thermosphacta ATCC 11509	BHI	26-30 C, aerobic
Lactobacillus acidophilus ATCC 4356	MRS	37°C, aerobic
Lactococcus cremoris ATCC 19257	BHI	26°C, aerobic
Lactobacillus casei ATCC 334	MRS	37°C, aerobic
Pediococcus pentosaceus ATCC33316	MRS	37°C, aerobic
Carnobacterium divergence ATCC 35677	TSB	30°C, aerobic
Pseudomonas aeruginosa ATCC 15442	TSB	37°C, aerobic
Escherichia Coli ATCC 35150	TSB	37°C, aerobic
Aeromonas hydrophila ATCC 7966	NB	30°C, aerobic
Klebsiella pneumonae ATCC13883	NB	37°C, aerobic
Campylobacter coli 2020/0011	TSB	37°C, microaerophilic
Enterobacter aerogenes ATCC 13048	NB	30°C, aerobic
Salmonella enteria Minnesota ATCC 9700	NB	37°C, aerobic
Salmonella enterica Typhimurium ATCC 14028	NB	37°C, aerobic

1: According to ATCC bacterial culture guideline.
